# Evolutionary implications of inversions that have caused intra-strand parity in DNA

**DOI:** 10.1186/1471-2164-8-160

**Published:** 2007-06-11

**Authors:** Kohji Okamura, John Wei, Stephen W Scherer

**Affiliations:** 1 The Centre for Applied Genomics, Program in Genetics and Genome Biology, The Hospital for Sick Children, MaRS Centre, Toronto, Ontario, Canada; 2 Department of Molecular and Medical Genetics, University of Toronto, Toronto, Ontario, Canada

## Abstract

**Background:**

Chargaff's rule of DNA base composition, stating that DNA comprises equal amounts of adenine and thymine (%A = %T) and of guanine and cytosine (%C = %G), is well known because it was fundamental to the conception of the Watson-Crick model of DNA structure. His second parity rule stating that the base proportions of double-stranded DNA are also reflected in single-stranded DNA (%A = %T, %C = %G) is more obscure, likely because its biological basis and significance are still unresolved. Within each strand, the symmetry of single nucleotide composition extends even further, being demonstrated in the balance of di-, tri-, and multi-nucleotides with their respective complementary oligonucleotides.

**Results:**

Here, we propose that inversions are sufficient to account for the symmetry within each single-stranded DNA. Human mitochondrial DNA does not demonstrate such intra-strand parity, and we consider how its different functional drivers may relate to our theory. This concept is supported by the recent observation that inversions occur frequently.

**Conclusion:**

Along with chromosomal duplications, inversions must have been shaping the architecture of genomes since the origin of life.

## Background

The most famous of Chargaff's rules is that in DNA, the proportion of A equals that of T, and C that of G [1]. This nucleotide balance is governed by complementary base-pairing rules fundamental to the structure of the double helix [2]. Astonishingly, the nucleotides retain almost the same equality balance in either of the two single strands of DNA [3] and this phenomenon is sometimes named Chargaff's second parity rule [4-10]. Table 1 provides an illustration, with analysis of large contiguous segments from each human chromosome.

**Table 1:** Mononucleotide content in contiguous single-stranded DNA scaffolds from each human chromosome *

Chromosome	Accession number	Length	%A	%T	%C	%G
1	NT_032977	73,835,825	29.72	29.69	20.33	20.27
2	NT_005403	84,213,157	30.60	30.68	19.34	19.38
3	NT_005612	100,530,253	30.51	30.53	19.46	19.49
4	NT_016354	92,123,751	31.34	31.33	18.64	18.69
5	NT_006576	46,378,398	30.45	30.31	19.62	19.62
6	NT_025741	61,645,385	30.84	30.86	19.16	19.14
7	NT_007933	64,426,257	30.43	30.39	19.62	19.56
8	NT_008046	57,155,273	30.21	30.04	19.89	19.86
9	NT_008470	40,394,265	28.72	28.72	21.27	21.28
10	NT_030059	44,617,998	29.12	29.30	20.80	20.77
11	NT_009237	49,571,094	29.57	29.70	20.36	20.37
12	NT_029419	38,648,979	30.06	30.01	19.96	19.97
13	NT_024524	67,740,325	30.97	30.93	19.06	19.04
14	NT_026437	88,290,585	29.44	29.67	20.42	20.47
15	NT_010194	53,619,965	29.06	28.82	21.11	21.01
16	NT_010498	42,003,582	28.32	28.31	21.66	21.70
17	NT_010783	24,793,602	28.22	28.25	21.76	21.76
18	NT_010966	33,548,238	30.34	30.23	19.73	19.71
19	NT_011109	31,383,029	26.25	26.32	23.68	23.76
20	NT_011362	26,144,333	27.26	27.56	22.57	22.61
21	NT_011512	28,617,429	30.57	30.31	19.60	19.52
22	NT_011520	23,276,302	26.33	26.29	23.72	23.67
X	NT_011651	36,813,576	31.07	31.36	18.74	18.82
Y	NT_011875	10,002,238	30.43	30.52	19.35	19.70
mtDNA	NC_001807	16,571	30.86	24.66	31.33	13.16

* The longest contig was chosen from each human chromosome.

When there is no bias in mutation and selection between complementary strands, base substitution may explain the parity phenomenon [11,12]. In fact, strand bias has been demonstrated with mutational skews between the two strands, which causes deviation from parity [13,15]. Bacterial origins of replication were successfully identified by the distribution of such skews [16,17]. The strand bias of mutations, which can be associated with direction of transcription, is also found in mammalian genomes [18,19]. In spite of these anomalies, any violation of the second parity phenomena is generally small in magnitude [8,20].

**Figure 1:**
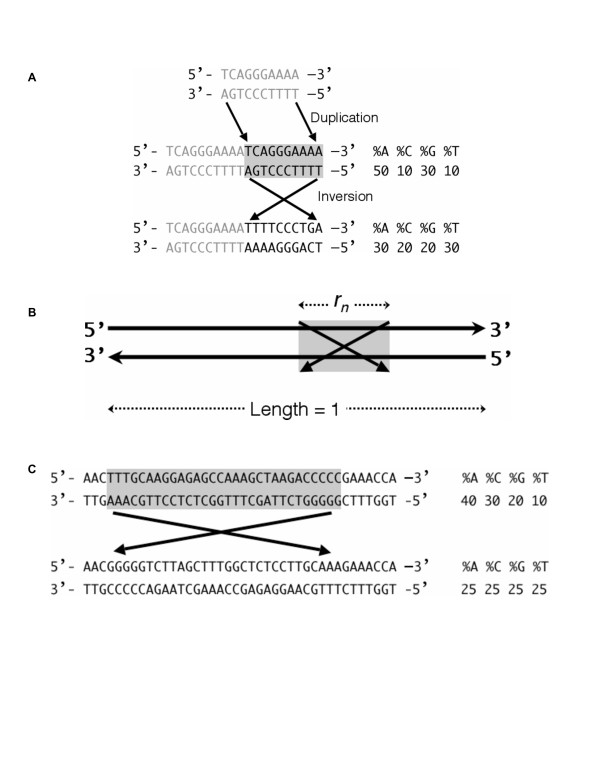
**Inversions as an explanation for intra-strand parity**. **A**, Duplication followed by inversion. If a double-stranded DNA, shown in gray, undergoes duplication and inversion, then the resulting molecule precisely demonstrates the strand parity (both within and between strands). **B**, A mathematical explanation of intra-strand parity. The *n*th inversion is illustrated by a box with crossed bars and *r*_*n *_is the relative length of the inversion within a total fragment of length = 1. Ultimately both *A*_*n *_and *T*_*n *_converge to the average of their initial frequencies. See Methods for details. Although a linear double-stranded DNA is shown, this could also be circular. **C**, A small number of inversions can cause DNA to follow the intra-strand parity. A 40-bp double-stranded DNA fragment in the human mtDNA (position 1875–1914 in accession number NC_001807) is shown, along with the outcome of a single artificial inversion, which has homogenized the contents of the two strands.

Although different explanations for this parity phenomenon have been put forth, such as intra-strand base pairing [6], a simpler explanation for the rule may be DNA duplication and inversion [4,8,10]. If double-stranded DNA of any composition undergoes duplication followed by an inversion of the duplicated region, then each strand of the resulting DNA molecule would precisely satisfy Chargaff's second parity rule, so that %A = %T and %C = %G (Fig. 1A).

Not only single nucleotides but also oligonucleotides up to 30 nucleotides (nt) in length can demonstrate the parity phenomenon within strands [5,7,8]. In other words, the frequency of a particular oligonucleotide is approximately equal to that of its reverse complementary sequence in the same strand. Since DNA strands are complementary, the frequency of a particular oligonucleotide in one strand approximates that in the opposite strand. Hence, this double-stranded DNA characteristic can also be called "symmetry of complementary DNA strands" [5,8]. Chargaff's second parity rule ordinarily considers only mononucleotides, which have been extensively studied. However, since a single nucleotide could be deemed a one-nt oligonucleotide, it is plausible that addressing the symmetry of oligonucleotides (high-order strand symmetry) is a more general way of assessing biological meaning. Hereafter, we designate this comprehensive symmetry as "intra-strand parity" and attempt to explain it based on the mechanism of chromosomal inversion. Single nucleotide mutations may be considered to explain mononucleotide parity within strands [11,12] but have not been effective to explain the extended parity of oligonucleotides [8].

## Results

We propose that inversion events (with or without underlying duplications) might be a sufficient mechanism to explain the phenomenon. To test this, we consider a double-stranded DNA molecule without intra-strand parity but which is long enough to undergo various (stochastic) inversions (Fig. 1B). *A*_*n *_and *T*_*n *_are defined as the frequency of any particular oligonucleotide sequence and its reverse complementary sequence, respectively, in the same strand after *n *inversions (*n *> 0). *A*_*0 *_(0 <*A*_*0*_ < 1) is the initial frequency of any particular oligonucleotide sequence (which can also be a mononucleotide) in the upper strand. *T*_*0 *_(0 <* T*_*0 *_< 1) is the initial frequency of its reverse complementary sequence in the same strand. If we define *r*_*n *_(0 <* r*_*n *_<< 1) as the relative length of the *n*th inversion (Fig. 1B), we obtain these two equations.

*A*_*n *_= *A*_*n*-1 _- *r*_*n*_(*A*_*n*-1 _- *T*_*n*-1_)

*T*_*n *_= *T*_*n*-1 _- *r*_*n*_(*T*_*n*-1 _- *A*_*n*-1_)

Equations (1) and (2) mean that an inversion changes *A*_*n *_and *T*_*n *_toward *T*_*n *_and *A*_*n*_, respectively. When the whole sequence is long enough, *r*_*n *_is close to 0. Nevertheless, whatever the size of the inverted region examined, any oligonucleotide sequence will eventually be homogenized between two strands. In other words, *A*_*n *_and *T*_*n *_ultimately converge to be equal to each other, regardless of *r*_*n*_, as long as *r*_*n *_is stochastic (see mathematical derivation in Methods).

lim⁡n→∞An=lim⁡n→∞Tn=A0+T02
 MathType@MTEF@5@5@+=feaafiart1ev1aaatCvAUfKttLearuWrP9MDH5MBPbIqV92AaeXatLxBI9gBaebbnrfifHhDYfgasaacH8akY=wiFfYdH8Gipec8Eeeu0xXdbba9frFj0=OqFfea0dXdd9vqai=hGuQ8kuc9pgc9s8qqaq=dirpe0xb9q8qiLsFr0=vr0=vr0dc8meaabaqaciaacaGaaeqabaqabeGadaaakeaadaWfqaqaaiGbcYgaSjabcMgaPjabc2gaTbWcbaGaemOBa4MaeyOKH4QaeyOhIukabeaakiabdgeabnaaBaaaleaacqWGUbGBaeqaaOGaeyypa0ZaaCbeaeaacyGGSbaBcqGGPbqAcqGGTbqBaSqaaiabd6gaUjabgkziUkabg6HiLcqabaGccqWGubavdaWgaaWcbaGaemOBa4gabeaakiabg2da9maalaaabaGaemyqae0aaSbaaSqaaiabicdaWaqabaGccqGHRaWkcqWGubavdaWgaaWcbaGaeGimaadabeaaaOqaaiabikdaYaaaaaa@4CDA@

Equation (3) is a mathematical explanation of intra-strand parity based on our hypothesis that inversions are sufficient to cause any DNA segment conform to parity. In this way, the vast majority of naturally occurring DNA molecules (chromosomes) will evolve to intra-strand parity via many inversions. Those few that deviate, such as mitochondrial DNA (mtDNA) [8,9,17], will have special properties (see below). We presume that any DNA can be made to evolve to intra-strand parity through a process of inversions, and that deviations from parity have been rare in evolution. Inversions must have been occurring as genomes of ancestral organisms were growing in complexity with the acquisition or creation of new genes.

The insertion of repetitive sequences was proposed to be a possible source underlying parity [8,10]. However, removing apparent repeats from the human and other genomes prior to analysis (see Methods) did not alter the symmetry characteristics of the remaining sequences. (An example of a 28.6-Mb contig from human chromosome 21 is shown in Table 2). Therefore, it is unlikely that insertion of such sequences accounts for the intra-strand parity, either in humans or organisms that have fewer repetitive sequences in their genomes.

**Table 2:** Dinucleotide frequencies in a human genomic contig without repetitive sequences *

Dinucleotide	Frequency	Difference	Frequency	Dinucleotide
AA	0.10956	0.00084	0.10872	TT
AC	0.04992	0.00047	0.04945	GT
AG	0.06718	0.00016	0.06702	CT
AT	0.08639	0.00000	0.08639	AT
CA	0.07012	0.00072	0.06940	TG
CC	0.04309	0.00027	0.04282	GG
CG	0.00781	0.00000	0.00781	CG
CT	0.06702	0.00016	0.06718	AG
GA	0.05869	0.00008	0.05876	TC
GC	0.03630	0.00000	0.03630	GC
GG	0.04282	0.00027	0.04309	CC
GT	0.04945	0.00047	0.04992	AC
TA	0.07474	0.00000	0.07474	TA
TC	0.05876	0.00008	0.05869	GA
TG	0.06940	0.00072	0.07012	CA
TT	0.10872	0.00084	0.10956	AA
Total	1.00000		1.00000	Total

* See text and Methods.

**Figure 2:**
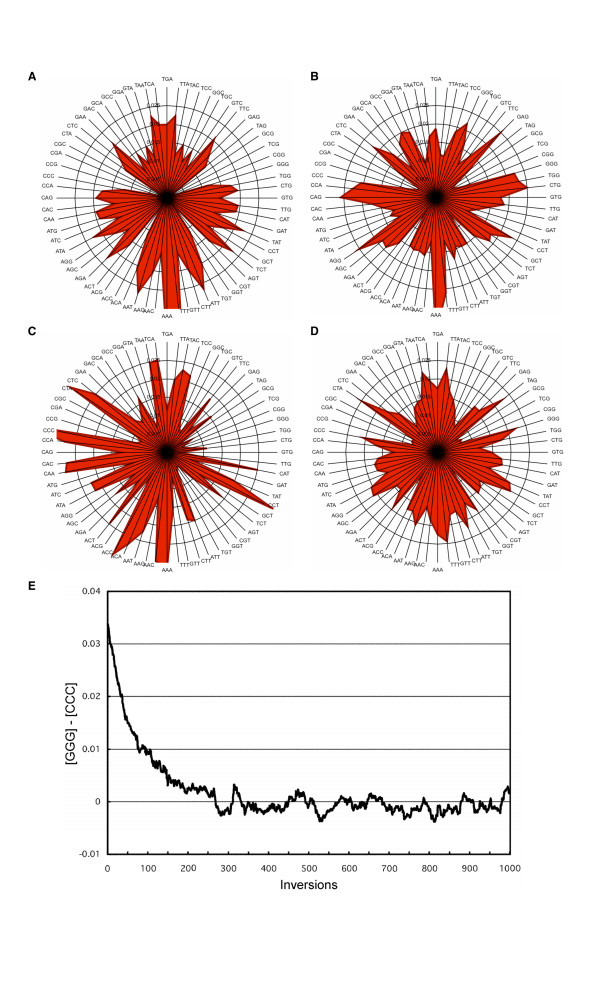
**Intra-strand parity visually represented by radar charts**. Frequencies of trinucleotides in various DNA sequences are shown here. Each trinucleotide is sorted alphabetically from bottom to top (left side). The corresponding complementary trinucleotides are arranged across to the right. **A**, Radar chart representing a fully sequenced contig (NT_010966, 33,548,238 bp) of human chromosome 18. This contig is continuous and does not include any annotated gaps or ambiguous nucleotides. The symmetrical chart shows the equal frequencies of specific oligonucleotides and their reverse complementary oligonucleotides. The high frequencies of poly-A and poly-T, which might be, in part, traces of retrotranspositions of poly-A^+ ^mRNA, and the deficiencies of trinucleotides that contain the CpG dinucleotide make the stalk and four grooves, respectively, of the "maple leaf" shape. (The shapes vary slightly based on the genome sequence analyzed, but the general symmetry is maintained). **B**, The genomic sequence of the *p53 *(*TP53*) locus (U94788, 20,303 bp). The symmetry is roughly retained in sequences as short as 20 kb in length. The protein-coding sequences occupy 5.8% of this locus. This chart also suggests that transcriptional asymmetry is small in magnitude. **C**, Human mtDNA. The asymmetry illustrates that this DNA does not show intra-strand parity. **D**, Human mtDNA after inversion *in silico*. It becomes symmetrical, demonstrating that inversions can change a sequence to create the parity. In this case, each *r*_*n *_approximates to 1/16.6. This also demonstrates that only 1/(2*r*_*ave*_) inversions (eight inversions in this case) are enough to make a sequence conform to parity. **E**, The difference of frequencies of GGG and CCC ([GGG] - [CCC]) in human mtDNA approaches 0 by *in silico *random inversions. In this analysis, for simplicity, the size of each inversion was fixed to 100 bp. In human mtDNA, GGG and CCC have the largest difference of frequencies among all trinucleoties (see Fig. 2C).

We employ radar charts to allow simple visual perception of the high-order symmetry and asymmetry of exemplary DNAs (Fig. 2). Mitochondria are thought to have been derived from bacteria [21]. Mammalian mtDNA (Fig 2C) is an exception that does not demonstrate intra-strand parity [8,9,17] whereas mtDNAs from plants and lower eukaryotes do. Mammalian mtDNA may have gradually deviated from its ancestral form [9]. The small circular size, its unique replication mechanism [22], and extra-nuclear localization could introduce different selective pressures against tolerance of inversions and thus deviation from the more general observation of intra-strand parity.

The mammalian mtDNA offers a natural source of sequence sufficiently deviating from parity to allow us to further test our mathematical explanation. We produced *in silico *semi-random inversions in human mtDNA. As few as eight 1-kb regularly-distributed inversions (see Methods) would be sufficient to homogenize the two strands of the 16.6-kb mtDNA and create intra-strand parity (Fig. 2D). We also depict a hypothetical inversion in the mtDNA to show the potential for rapid homogenization (Fig. 1C).

Although the lack of intra-strand parity in mammalian mtDNA could be ascribed to its small length, other loci of comparable length (*e.g*. the *TP53 *gene, Fig. 2B) do adhere to parity. Unlike other mtDNAs, those of mammals have no intergenic segments and have only one regulatory region per strand. Moreover, unlike among nuclear genomes, the order and direction of genes – as well as biased gene density between the two strands – are strictly conserved among mammalian species [23]. Therefore, it seems that the configuration is already fixed, and that inversions are not tolerated in mammalian mtDNA.

## Discussion

The ubiquity of inversions suggests that they had some advantage in natural selection. Duplications are thought to play an important role in creating genetic variety [24], however, some duplications are deleterious for organisms, due to sudden increases of gene dosage. To avoid being negatively selected, one of the duplicated copies could undergo mutation such as deletion. Inversions or interchromosomal rearrangements could render the duplicated gene nonfunctional due to its release from interaction with its promoter or other regulatory elements. This may be one reason why many inverted and interchromosomal segmental duplications are found in the human genome [25,26]. An approximately symmetrical gene distribution between the two strands may have been brought about by these rearrangements [27].

In some cases, a rearranged genome might confer positive selection. Although we can find syntenic regions among vertebrates, chromosomal organizations can be quite different among species. This suggests an advantage for evolution or speciation. Recently, the importance of gene order and gene position in the three-dimensional nucleus has been suggested [28]. It is likely that genomes continually undergo rearrangement toward optimal positions for each gene and each gene cluster. Our group showed an unexpectedly large number of inversions (from 23 bp to 62 Mb in size) between human and chimpanzee genomes [29], species which diverged only six million years ago. Although most may be selectively neutral, some likely were selected for, and contributed to the speciation. Many more inversions may also have occurred and may have been negatively selected. Inversions can also give rise to new transcripts, some of which will be selected for and become new genes. We identified hybrid transcripts of the *AZGP1 *and *GJE1 *genes on human chromosome 7 (manuscript in preparation) and are intrigued that the orthologues of these genes in non-primate mammals reside in a head-to-head manner. It is likely that the common ancestor of primates underwent inversion of the *AZGP1 *gene to produce the hybrid transcripts, creating an opportunity for primate diversity.

## Conclusion

In summary, we propose that the relatively frequent occurrence and accumulation of inversions in genomes may be a major contributor to the phenomenon of intra-strand parity. Whereas single base substitutions might explain Chargaff's second parity rule at the level of mononucleotides, they can explain neither the high-order intra-strand parity nor the exceptional deviation of mammalian mtDNAs. In contrast, inversion events are not limited by size and can involve millions of bases of sequence. Other mechanisms may have contributed to some extent; nevertheless, they are not necessary to account for intra-strand parity if inversions are considered.

Inversions are one process contributing to genome evolution that allow for rearrangement toward optimal position, order, and orientation of genes and regulatory elements, and for escape from deleterious effects caused, for example, by some duplications. Although we acknowledge the possibility of preferential sites, inversions occur randomly as shown in our mathematical explanation. Many of these are expected to be deleterious and would presumably be selected against, but others should be neutral or positively selected and could therefore become fixed in the genome [30]. Quantitative estimation of inversion using genomic sequences of extant organisms is unfortunately meaningless, as it cannot account for those events lost to natural selection. Further, inversions must have contributed to the basic character of DNA sequences since the origin of life. There are now substantial data supporting the frequency of inversions within genomes of a variety of organisms, including plants, insects and primates [29-33], and these observable events are but the tip of the iceberg. Chromosomal rearrangements such as inversions reduce the rate of meiotic recombination between homologous chromosomes, with subsequent reproductive isolation [34]. Moreover, in these regions, mutations tend to be positively selected to give rise to speciation [35]. Ohno's seminal work [24] and that of others have emphasized the importance of duplications in evolution. Our suppositions further these ideas, in particular suggesting how inversions and duplications can complement each other to yield the properties of extant genomes.

## Methods

### Calculation of frequencies of oligonucleotides

The genomic sequences (human contigs, the *TP53 *gene, and the mtDNA sequence) were downloaded from NCBI (Build 36). Calculation of frequencies of oligonucleotides (including mononucleotides) was performed using Perl scripts, which are available upon request. The "plus" strand, which is stored in the database, was analyzed. We generated sequence free of repetitive elements using RepeatMasker with which 46.4% of the 28,617,429 nucleotides were masked. The coordinates of the eight 1-kb regularly-scattered *in silico *inversions were 1001–2000, 3001–4000, 5001–6000, 7001–8000, 9001–10000, 11001–12000, 13001–14000, and 15001–16000 in NC_001807.

### Mathematical derivation

For the frequency of a particular oligonucleotide *A*_*n *_(*n *> 0), via the *n*th inversion, (1 - *r*_*n*_) *A*_*n*-*1 *_remains; *r*_*n*_*A*_*n*-*1 *_decreases; *r*_*n*_*T*_*n*-*1 *_increases if we suppose the distribution of contents is even in the whole sequence. In this way, the two recurrence formulas (1) and (2) are derived (see text). The following equations are obtained by adding equations (1) and (2).

*A*_*n *_+ *T*_*n *_= *A*_*n*-1 _+ *T*_*n*-1_

*A*_*n *_+ *T*_*n *_= *A*_0 _+ *T*_0_

These mean that inversions do not change the sum of the two frequencies. Using (5), other forms of (1) and (2) are derived.

*A*_*n *_= (1 - 2*r*_*n*_)*A*_*n*-1 _+ *r*_*n*_(*A*_0 _+ *T*_0_)

*T*_*n *_= (1 - 2*r*_*n*_)*T*_*n*-1 _+ *r*_*n*_(*A*_0 _+ *T*_0_)

When we subtract (*A*_*0 *_+ *B*_*0*_)/2 from (6) and define *B*_*n*_, (9) is derived.

Bn=An−A0+B02
 MathType@MTEF@5@5@+=feaafiart1ev1aaatCvAUfKttLearuWrP9MDH5MBPbIqV92AaeXatLxBI9gBaebbnrfifHhDYfgasaacH8akY=wiFfYdH8Gipec8Eeeu0xXdbba9frFj0=OqFfea0dXdd9vqai=hGuQ8kuc9pgc9s8qqaq=dirpe0xb9q8qiLsFr0=vr0=vr0dc8meaabaqaciaacaGaaeqabaqabeGadaaakeaacqWGcbGqdaWgaaWcbaGaemOBa4gabeaakiabg2da9iabdgeabnaaBaaaleaacqWGUbGBaeqaaOGaeyOeI0YaaSaaaeaacqWGbbqqdaWgaaWcbaGaeGimaadabeaakiabgUcaRiabdkeacnaaBaaaleaacqaIWaamaeqaaaGcbaGaeGOmaidaaaaa@3A31@

Bn=(1−2rn)Bn−1=(1−2r1)(1−2r2)(1−2r3)...(1−2rn−1)B0=B0∏k=1n−1(1−2rk)=A0−T02∏k=1n−1(1−2rk)
 MathType@MTEF@5@5@+=feaafiart1ev1aaatCvAUfKttLearuWrP9MDH5MBPbIqV92AaeXatLxBI9gBaebbnrfifHhDYfgasaacH8akY=wiFfYdH8Gipec8Eeeu0xXdbba9frFj0=OqFfea0dXdd9vqai=hGuQ8kuc9pgc9s8qqaq=dirpe0xb9q8qiLsFr0=vr0=vr0dc8meaabaqaciaacaGaaeqabaqabeGadaaakeaafaqaaeabbaaaaeaacqWGcbGqdaWgaaWcbaGaemOBa4gabeaakiabg2da9iabcIcaOiabigdaXiabgkHiTiabikdaYiabdkhaYnaaBaaaleaacqWGUbGBaeqaaOGaeiykaKIaemOqai0aaSbaaSqaaiabd6gaUjabgkHiTiabigdaXaqabaaakeaacqGH9aqpcqGGOaakcqaIXaqmcqGHsislcqaIYaGmcqWGYbGCdaWgaaWcbaGaeGymaedabeaakiabcMcaPiabcIcaOiabigdaXiabgkHiTiabikdaYiabdkhaYnaaBaaaleaacqaIYaGmaeqaaOGaeiykaKIaeiikaGIaeGymaeJaeyOeI0IaeGOmaiJaemOCai3aaSbaaSqaaiabiodaZaqabaGccqGGPaqkcqGGUaGlcqGGUaGlcqGGUaGlcqGGOaakcqaIXaqmcqGHsislcqaIYaGmcqWGYbGCdaWgaaWcbaGaemOBa4MaeyOeI0IaeGymaedabeaakiabcMcaPiabdkeacnaaBaaaleaacqaIWaamaeqaaaGcbaGaeyypa0JaemOqai0aaSbaaSqaaiabicdaWaqabaGcdaqeWbqaaiabcIcaOiabigdaXiabgkHiTiabikdaYiabdkhaYnaaBaaaleaacqWGRbWAaeqaaOGaeiykaKcaleaacqWGRbWAcqGH9aqpcqaIXaqmaeaacqWGUbGBcqGHsislcqaIXaqma0Gaey4dIunaaOqaaiabg2da9maalaaabaGaemyqae0aaSbaaSqaaiabicdaWaqabaGccqGHsislcqWGubavdaWgaaWcbaGaeGimaadabeaaaOqaaiabikdaYaaadaqeWbqaaiabcIcaOiabigdaXiabgkHiTiabikdaYiabdkhaYnaaBaaaleaacqWGRbWAaeqaaOGaeiykaKcaleaacqWGRbWAcqGH9aqpcqaIXaqmaeaacqWGUbGBcqGHsislcqaIXaqma0Gaey4dIunaaaaaaa@8C53@

Using -1 << 1 - 2*r*_*k *_< 1 (0 <*r*_*k *_<< 1), lim⁡n→∞Bn=A0−T02lim⁡n→∞∏k=1n−1(1−2rk)=0
 MathType@MTEF@5@5@+=feaafiart1ev1aaatCvAUfKttLearuWrP9MDH5MBPbIqV92AaeXatLxBI9gBaebbnrfifHhDYfgasaacH8akY=wiFfYdH8Gipec8Eeeu0xXdbba9frFj0=OqFfea0dXdd9vqai=hGuQ8kuc9pgc9s8qqaq=dirpe0xb9q8qiLsFr0=vr0=vr0dc8meaabaqaciaacaGaaeqabaqabeGadaaakeaadaWfqaqaaiGbcYgaSjabcMgaPjabc2gaTbWcbaGaemOBa4MaeyOKH4QaeyOhIukabeaakiabdkeacnaaBaaaleaacqWGUbGBaeqaaOGaeyypa0ZaaSaaaeaacqWGbbqqdaWgaaWcbaGaeGimaadabeaakiabgkHiTiabdsfaunaaBaaaleaacqaIWaamaeqaaaGcbaGaeGOmaidaamaaxababaGagiiBaWMaeiyAaKMaeiyBa0galeaacqWGUbGBcqGHsgIRcqGHEisPaeqaaOWaaebCaeaacqGGOaakcqaIXaqmcqGHsislcqaIYaGmcqWGYbGCdaWgaaWcbaGaem4AaSgabeaakiabcMcaPiabg2da9iabicdaWaWcbaGaem4AaSMaeyypa0JaeGymaedabaGaemOBa4MaeyOeI0IaeGymaedaniabg+Givdaaaa@5B54@.

Therefore, lim⁡n→∞An=lim⁡n→∞Bn+A0+T02=A0+T02
 MathType@MTEF@5@5@+=feaafiart1ev1aaatCvAUfKttLearuWrP9MDH5MBPbIqV92AaeXatLxBI9gBaebbnrfifHhDYfgasaacH8akY=wiFfYdH8Gipec8Eeeu0xXdbba9frFj0=OqFfea0dXdd9vqai=hGuQ8kuc9pgc9s8qqaq=dirpe0xb9q8qiLsFr0=vr0=vr0dc8meaabaqaciaacaGaaeqabaqabeGadaaakeaadaWfqaqaaiGbcYgaSjabcMgaPjabc2gaTbWcbaGaemOBa4MaeyOKH4QaeyOhIukabeaakiabdgeabnaaBaaaleaacqWGUbGBaeqaaOGaeyypa0ZaaCbeaeaacyGGSbaBcqGGPbqAcqGGTbqBaSqaaiabd6gaUjabgkziUkabg6HiLcqabaGccqWGcbGqdaWgaaWcbaGaemOBa4gabeaakiabgUcaRmaalaaabaGaemyqae0aaSbaaSqaaiabicdaWaqabaGccqGHRaWkcqWGubavdaWgaaWcbaGaeGimaadabeaaaOqaaiabikdaYaaacqGH9aqpdaWcaaqaaiabdgeabnaaBaaaleaacqaIWaamaeqaaOGaey4kaSIaemivaq1aaSbaaSqaaiabicdaWaqabaaakeaacqaIYaGmaaaaaa@5400@.

Similarly, lim⁡n→∞Tn=A0+T02
 MathType@MTEF@5@5@+=feaafiart1ev1aaatCvAUfKttLearuWrP9MDH5MBPbIqV92AaeXatLxBI9gBaebbnrfifHhDYfgasaacH8akY=wiFfYdH8Gipec8Eeeu0xXdbba9frFj0=OqFfea0dXdd9vqai=hGuQ8kuc9pgc9s8qqaq=dirpe0xb9q8qiLsFr0=vr0=vr0dc8meaabaqaciaacaGaaeqabaqabeGadaaakeaadaWfqaqaaiGbcYgaSjabcMgaPjabc2gaTbWcbaGaemOBa4MaeyOKH4QaeyOhIukabeaakiabdsfaunaaBaaaleaacqWGUbGBaeqaaOGaeyypa0ZaaSaaaeaacqWGbbqqdaWgaaWcbaGaeGimaadabeaakiabgUcaRiabdsfaunaaBaaaleaacqaIWaamaeqaaaGcbaGaeGOmaidaaaaa@400A@.

## Authors' contributions

KO conceived the study, performed the computational analyses, mathematical derivation, and drafted the manuscript. JW participated in the coordination of the study and performed the computational analyses. SWS participated in the design and coordination of the study and helped draft the manuscript. All authors read and approved the final manuscript.

## References

[B1] Chargaff E (1951). Structure and function of nucleic acids as cell constituents. Fed Proc.

[B2] Watson JD, Crick FH (1953). Molecular structure of nucleic acids: a structure for deoxyribose nucleic acid. Nature.

[B3] Rudner R, Karkas JD, Chargaff E (1968). Separation of *B. subtilis *DNA into complementary strands. 3. Direct analysis. Proc Natl Acad Sci USA.

[B4] Fickett JW, Torney DC, Wolf DR (1992). Base compositional structure of genomes. Genomics.

[B5] Prabhu VV (1993). Symmetry observations in long nucleotide sequences. Nucleic Acids Res.

[B6] Forsdyke DR, Mortimer JR (2000). Chargaff's legacy. Gene.

[B7] Qi D, Cuticchia AJ (2001). Compositional symmetries in complete genomes. Bioinformatics.

[B8] Baisnée PF, Hampson S, Baldi P (2002). Why are complementary DNA strands symmetric?. Bioinformatics.

[B9] Mitchell D, Bridge R (2006). A test of Chargaff's second rule. Biochem Biophys Res Commun.

[B10] Albrecht-Buehler G (2006). Asymptotically increasing compliance of genomes with Chargaff's second parity rules through inversions and inverted transpositions. Proc Natl Acad Sci USA.

[B11] Sueoka N (1995). Intrastrand parity rules of DNA base composition and usage biases of synonymous codons. J Mol Evol.

[B12] Lobry JR (1995). Properties of a general model of DNA evolution under no-strand-bias conditions. J Mol Evol.

[B13] McLean MJ, Wolfe KH, Devine KM (1998). Base composition skews, replication orientation, and gene orientation in 12 prokaryote genomes. J Mol Evol.

[B14] Bell SJ, Forsdyke DR (1999). Deviations from Chargaff's second parity rule correlate with direction of transcription. J Theor Biol.

[B15] Daubin V, Perriere G (2003). G+C3 structuring along the genome: a common feature in prokaryotes. Mol Biol Evol.

[B16] Nikolaou C, Almirantis Y (2005). A study on the correlation of nucleotide skews and the positioning of the origin of replication: different modes of replication in bacterial species. Nucleic Acids Res.

[B17] Nikolaou C, Almirantis Y (2006). Deviations from Chargaff's second parity rule in organellar DNA insights into the evolution of organellar genomes. Gene.

[B18] Green P, Ewing B, Miller W, Thomas PJ, Green ED, NISC Comparative Sequencing Program (2003). Transcription-associated mutational asymmetry in mammalian evolution. Nat Genet.

[B19] Louie E, Ott J, Majewski J (2003). Nucleotide frequency variation across human genes. Genome Res.

[B20] Prescott DM, Dizick SJ (2000). A unique pattern of intrastrand anomalies in base composition of the DNA in hypotrichs. Nucleic Acids Res.

[B21] Fileé J, Forterre P (2005). Viral proteins functioning in organelles: a cryptic origin?. Trends Microbiol.

[B22] Clayton DA (1982). Replication of animal mitochondrial DNA. Cell.

[B23] Pääbo S, Thomas WK, Whitfield KM, Kumazawa Y, Wilson AC (1991). Rearrangements of mitochondrial transfer RNA genes in marsupials. J Mol Evol.

[B24] Ohno S (1970). Evolution by Gene and Genome Duplication.

[B25] Bailey JA, Gu Z, Clark RA, Reinert K, Samonte RV, Schwartz S, Adams MD, Myers EW, Li PW, Eichler EE (2002). Recent segmental duplications in the human genome. Science.

[B26] Cheung J, Estivill X, Khaja R, MacDonald JR, Lau K, Tsui LC, Scherer SW (2003). Genome-wide detection of segmental duplications and potential assembly errors in the human genome sequence. Genome Biol.

[B27] Dunham I, Shimizu N, Roe BA, Chissoe S, Hunt AR, Collins JE, Bruskiewich R, Beare DM, Clamp M, Smink LJ, Ainscough R, Almeida JP, Babbage A, Bagguley C, Bailey J, Barlow K, Bates KN, Beasley O, Bird CP, Blakey S, Bridgeman AM, Buck D, Burgess J, Burrill WD, O'Brien KP (1999). The DNA sequence of human chromosome 22. Nature.

[B28] Kosak ST, Groudine M (2004). Gene order and dynamic domains. Science.

[B29] Feuk L, MacDonald JR, Tang T, Carson AR, Li M, Rao G, Khaja R, Scherer SW (2005). Discovery of human inversion polymorphisms by comparative analysis of human and chimpanzee DNA sequence assemblies. PLoS Genet.

[B30] Hoffmann AA, Sgrò CM, Weeks AR (2004). Chromosomal inversion polymorphisms and adaptation. Trends Ecol Evol.

[B31] Blanc G, Barakat A, Guyot R, Cooke R, Delseny M (2000). Extensive duplication and reshuffling in the *Arabidopsis *genome. Plant Cell.

[B32] Coluzzi M, Sabatini A, della Torre A, Di Deco MA, Petrarca V (2002). A polytene chromosome analysis of the *Anopheles gambiae *species complex. Science.

[B33] Tuzun E, Sharp AJ, Bailey JA, Kaul R, Morrison VA, Pertz LM, Haugen E, Hayden H, Albertson D, Pinkel D, Olson MV, Eichler EE (2005). Fine-scale structural variation of the human genome. Nat Genet.

[B34] Rieseberg LH (2001). Chromosomal rearrangements and speciation. Trends Ecol Evol.

[B35] Navarro A, Barton NH (2003). Chromosomal speciation and molecular divergence – accelerated evolution in rearranged chromosomes. Science.

